# Return to work following primary total hip arthroplasty: a systematic review and meta-analysis

**DOI:** 10.1186/s13018-023-03578-y

**Published:** 2023-02-12

**Authors:** Mohammad Soleimani, Mazyar Babagoli, Soroush Baghdadi, Peyman Mirghaderi, Yousef Fallah, Mehrdad Sheikhvatan, Seyyed Hossein Shafiei

**Affiliations:** 1 Orthopedic Surgery Research Centre( OSRC), Sina University Hospital, Tehran University of Medical Sciences, Tehran, Iran; 2 Pediatric Orthopaedic Surgery Department, Montefiore Medical Center, New York, USA

**Keywords:** Total hip arthroplasty, Primary THA, Return to work, Activities, Total joint arthroplasty

## Abstract

**Background:**

Total hip arthroplasty (THA) is increasingly common in younger patients, who are more likely to be working preoperatively. There is a need for an updated review of the literature regarding the rate and time to return to work (RTW), which is important when counseling patients, and also from an economic standpoint.

**Methods:**

A systematic review and meta-analysis of the literature was performed on January 20, 2022, and studies reporting the rate and/or time to RTW after THA were included. Two authors independently selected relevant papers. RTW was extracted and analyzed using fixed-effects or random-effects models where appropriate.

**Results:**

A total of 48 studies were included in the final analysis. We found that 70.7% of patients were working after primary THA. Among patients who were working before surgery, this rate increases to 87.9%, while 28.1% of patients who were not working preoperatively started working after surgery. Younger patients were more likely to RTW, while patients with a physically demanding job were less likely to RTW. Minimally invasive techniques were reported to yield a higher rate of RTW and an earlier time to RTW.

**Conclusion:**

We found that the majority of patients return to work after THA, and some patients are able to start working after surgery. Compared to previous reviews, patients seem to have a higher rate and earlier RTW. The overall trend of the literature suggests that patients are returning to work earlier and at a higher rate compared to previous reviews.

## Introduction

As the volume of total hip arthroplasties (THA) is increasing globally, the number of younger patients is also growing. With the advent of modern surgical techniques and the increasing longevity of current designs, patients younger than 65 years are expected to constitute more than half of all arthroplasties in the USA by 2030 [1]. In addition, the number of THAs performed is projected to double during the same period. Therefore, functional outcomes of THA and the patients’ return to activities are an increasingly important metric in measuring success after hip reconstruction.

Data on return to daily activities after THA, including return to sports and return to work (RTW), are scarce in the literature. RTW after THA in patients of working age has a substantial economic impact on the patient, family, and the healthcare system, as well as the psychological and physical benefits to the individual [2]. Previous studies have been heterogeneous regarding the rate and timing of RTW, reporting a wide range from 25 to 122%, the latter indicating more patients being working at the follow-up [3]. Interestingly, more recent reports show a trend of an increasing rate of RTW, as well as a shorter duration of inability to work after surgery. The last study to systematically review the RTW data was performed in 2017, and considering the fast pace of the current joint literature and several more recent papers on this topic, there is a need for an updated review [3].

Therefore, this study was performed to systematically review the current joint literature to determine the rate of return to work after THA, time to RTW, and the potential predictors for successful RTW following THA. The results of this study would be important not only to surgeons and the healthcare system, but also to patients who will be directly affected by this outcome.

## Methods

The PRISMA (Preferred Reporting Items for Systematic Reviews and Meta-Analyses) guidelines were followed to perform this systematic review. The study protocol was also published on the international prospective register of systematic reviews, PROSPERO (record number CRD42022307385, available at https://www.crd.york.ac.uk/prospero/display_record.php?ID=CRD42022307385).

### Search strategy

We performed a systematic review on January 20, 2022. We searched PubMed MEDLINE, Embase, Scopus, Web of Science, and the Cochrane library. Search queries were personalized to follow each database’s rules and regulations, with the general term of ("Total Hip Replacement" OR "Total Hip Arthroplasty" OR "Hip Prosthesis Implantation") AND ("Return to Work" OR "Back to Work" OR "Return to occupation").

### PICO and inclusion/exclusion criteria

Clinical trials, retrospective, and prospective observational studies with a study population of adults were selected.

Our targeted population included the patients underwent total hip replacement (Patients and interventions), the outcomes compared between subgroups were the rate and time of returning to work and also the factors affecting return to work (comparison and outcome).

Exclusion criteria were: revision THA, those surgeries that were not primary were excluded from our studies, THA in combination with another surgical procedure was the other exclusion criteria, That is, only those studies were added that patients had undergone a total hip arthroplasty surgery and had not performed another surgery at the same time or in the same admission that would affect the results of the hip replacement surgery. Review articles, book chapters, case reports, and non-English studies were other exclusion criteria. With all these, there was no limit to the publication year of selected studies.

### Quality assessment

The MINORS (Methodological Index for Non-Randomized Studies) criteria was utilized to assess study quality. MINORS is a framework for scoring non-randomized studies such as observational and descriptive studies. MINORS includes 12 items graded from 0 to 2, with maximum scores of 16 for non-comparative studies and 24 for comparative studies. Higher scores indicate a higher quality of evidence [4]. Scores of 0–8 or 0–12 were considered low quality, 9–12 or 13–18 were deemed to be moderate quality, and 13–16 or 19–24 were regarded as high quality, respectively, for non-comparative and comparative studies (Appendix 1).

### Data extraction

EndNote version 20 (Clarivate, Philadelphia, PA) was used to screen the articles. Two reviewers (M.B. and M.S.) independently reviewed the titles and abstracts of each paper to select relevant papers. Discrepancies were addressed by a third author (P.M.). Data from the selected studies were collected by two reviewers (M.B. and M.S.). The course of each person's review was as follows: After extracting the articles from the databases introduced, in the screening stage the duplicated items were excluded, and then, the titles and summaries were checked. At this stage, a number of articles were selected for full-text review. In the review of the full text, the studies that did not investigate the desired outcomes were excluded. Finally, after these reviews, the remaining studies were selected for systematic review, while the studies that the reports of the results could be analyzed in terms of meta-analysis were also included in the meta-analysis cycle.

### Quality of the evidence (GRADE system)

GRADE is a system for assessing the quality of the evidence of each result in a review against eight criteria (including risk of bias, inconsistency, indirectness, imprecision, and publication bias). In the GRADE system, the quality of evidence for each outcome is graded as HIGH, MODERATE, LOW or VERY LOW. Quality of evidences assessment for this study was done separately the by two authors (M.S & M.B) and the final conclusion was made by evaluating their results.

### Statistical analysis

RTW data were pooled using Comprehensive Meta-Analysis (CMA) software (BioStat Inc., Englewood, NJ). Depending on the degree of statistical heterogeneity, either fixed-effects or random-effects models were used. Statistical heterogeneity was tested using the tau-squared test and I-squared test, that was completed with the *I*^2^ statistic, which quantifies the proportion of total variation across studies that is due to heterogeneity. A value of 0–25% indicates insignificant heterogeneity, while 26–50% low heterogeneity, 51–75% moderate heterogeneity and 76–100% high heterogeneity. This study calculated the pooled weighted event rates of RTW. The 95% confidence interval was used to calculate the mean prevalence. The funnel plot and Egger's regression with a 95% confidence interval were used to evaluate publication bias.

## Results

### Study characteristics

**Fig. 1:**
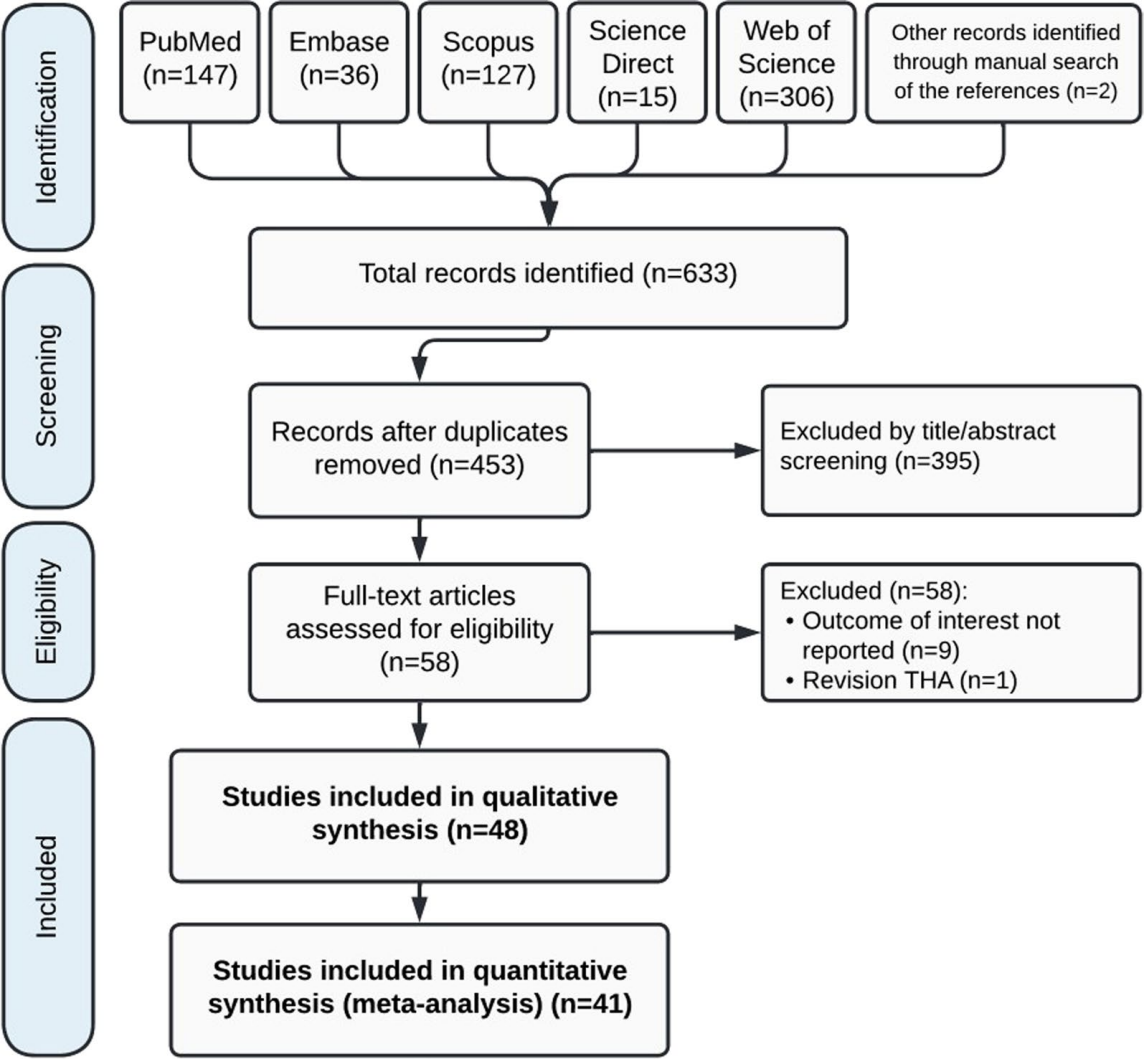
Flow diagram outlining the process of study selection. Adapted from the PRISMA (Preferred Reporting Items for Systematic Reviews and Meta-Analyses) statement

The initial search yielded 633 records. After multiple rounds of screening and exclusion, 48 articles were finally included in the qualitative synthesis (Fig. 1) [2, 5–52].

In the initial screening, 633 articles were selected, which reached 453 after removing duplicates, 395 were excluded from the study in the title and abstract review, and finally 58 studies were subjected to full-text review among these 9 articles excluded for not paying to the outcomes in question and one study due to not paying attention to the primary total hip arthroplasty (dealing with revision arthroplasty) were excluded from the study. Finally, 48 studies were selected and subjected to systematic review, and 41 of them had the desired characteristics for meta-analysis.

As shown in Table 3, the included studies had an average MINORS score of 14.8 ± 4.3 (range, 7–23), indicating a moderate quality of evidence. There were 11 studies with high quality, 36 with moderate quality, and 1 with low quality. The 48 included studies were published between 1965 and 2022, comprising a combined population of 9267 patients. The mean follow-up ranged from 6 weeks to 20.1 years. Table 1 summarizes the basic characteristics of these studies, and the outcomes of interest, including the RTW rate and time to RTW, are shown in Table 2.

**Table 1:** Characteristics of the studies included in this systematic review

Author	Study design	Study group	Patients	Male, female	Mean age	Follow-up
Al-Hourani [5]	Prospective cohort	Inclusion criteria: patients < 65 years of age. Exclusion criteria: patients undergoing bilateral TJA or revision procedures	91	42, 49	59	1 year
Anderson [6]	Retrospective cohort	Exclusion criteria: patients with any additional injuries, a hip fracture other than a subcapital or transcervical FNF, a non-displaced FNF, age less than 45 or greater than 65 were excluded	23	9, 14	58.5	2 years
Atkinson [7]	Retrospective cohort	Inclusion criteria: Patients with bilateral hip osteoarthritis	39	24, 15	62.87	34 months
Baldursson [8]	Retrospective cohort	Inclusion criteria: patients with ankylosing spondylitis	10	8, 2	32	3.8 years
Batra [9]	Prospective cohort	Inclusion criteria: driving patients, who didn't undergo for femur fractures and revision THAs	198	69, 129	69	8 weeks
Berger [10]	Prospective cohort	Inclusion criteria: patients between 40 and 75 years of age having primary THA who did not have a history of previous hip surgery. Exclusion criteria: Patients with a history (within 1 year) of myocardial infarction, pulmonary embolism, or anticoagulation therapy were excluded. In addition, patients with considerable obesity (body mass index more than 35) or with three or more important medical comorbidities, that were not controlled	100	74, 26	56	3 months
Boersma [11]	Prospective cohort	Inclusion criteria: patients between 18–63 years of age, had a paid job, were scheduled to undergo THA or TKA as a result of primary osteoarthritis	97	41, 56	56	12 months
Bohm [2]	Retrospective Cohort	Inclusion criteria: working aged patients	60	22, 24(missing)	51.35	1 year
Clyde [12]	Prospective Cohort	Inclusion criteria: patients 18 and older receiving Workers' Compensation at the time of their THA	43	31, 12		5.2 years
Danielsson [13]	Retrospective Cohort		30	10, 20	59	3–4 years
Drobniewski [14]	Retrospective Cohort	Inclusion criteria: women under the age of 59 years and men under the age of 64 years who underwent surgery due to advanced hip osteoarthritis	114	66, 48	51.23	12 months
Drobniewski [15]	Retrospective cohort	Inclusion criteria: Patients aged ≤ 30 years	87	34, 53	25.7	20.1 years
Goeb [16]	Prospective cohort	Inclusion criteria: Patients aged 35–70 years. Exclusion criteria: patients with inflammatory arthritis, osteoarthritis with multiple joint involvement such that other joints limit functional status and mobility, abnormal renal function, hypercoagulable state, body mass index > 40, previous ipsilateral femoral neck fracture, chronic opioid use, dementia, Alzheimer’s, neuromuscular conditions limiting ambulation, and patients who underwent contralateral THA in the prior 6 months	72	42, 30	60.4	6 weeks
Hauer [17]	Retrospective cohort	Inclusion criteria: patients below the retirement age (male < 65, female < 62) at the time of their THA, had a diagnosis of non-inflammatory arthritis (osteoarthritis, osteonecrosis, developmental dysplasia) and were at least 6 months post-surgery were included. Exclusion criteria: individuals who were fewer than 6 months post-surgery, had a one-stage bilateral THA or hip replacement due to a femoral neck fracture or a tumor	273	145, 128	53.9	30.9 months
He [18]	Retrospective cohort	Inclusion criteria: patients with ankylosing spondylitis aged between 18 to 65 years old Exclusion criteria: patients with a history of revision arthroplasty, other lower limb or spine surgery; patients with medical comorbidities, including severe lung, heart, or other diseases which could affect the work ability;	128	107, 21	40	12 months
Jensen [19]	Retrospective cohort	Inclusion criteria: patients with hip disease	387	139, 248	67	5 years
Johnsson [20]	Retrospective cohort	Inclusion criteria: patients who were below the age of 60 at surgery with primary arthrosis	118	76, 42	54	2 years
Kamp [21]	Prospective cohort	Inclusion criteria: patients who underwent THA for primary OSTEOARTHRITIS. preoperatively employed and aged 18–63 were included	100	46, 54	56	12 months
Kleim [51]	Cross-sectional	Inclusion criteria: Those who were 6 months to 3 years after surgery had a diagnosis of OA and were under the age of sixty at the time of their primary total hip arthroplasty (THA) were included. Only patients five or more years below the retirement age (65) were recruited	52	23, 29	52	22 months
Laasik [22]	Retrospective cohort	Inclusion criteria: patients employed for a minimum of 6 months in the participating organizations	408	110, 298	54.3	1 year
Latijnhouwers [23]	Retrospective cohort	Inclusion criteria: Data from patients that were scheduled for primary THA because of osteoarthritis	876	335, 541	68	12 months
Leichtenberg [24]	Prospective cohort	Inclusion criteria: patients with osteoarthritis. exclusion criteria: Patients with rheumatoid arthritis, a tumor, (hemi)paresis or amputation of the (lower) leg, and patients undergoing a hemiarthroplasty or revision THA or TKA	67	34, 33	56	12 months
Mangin [52]	Retrospective cohort	Inclusion criteria: patients aged < 65 years at the time of surgery. primary THA performed for orthopedic reasons (excluding trauma) and being in work during 2 years prior to surgery	72			1036 days
Mcgonagle [25]	Retrospective cohort	Inclusion criteria: patients aged ≤ 65 years at the time of surgery; and engaged in paid work in the 3 months prior to surgery. Exclusion criteria: revision hip/knee arthroplasty and bilateral concurrent hip/knee arthroplasty	58			6–12 months
Mikkelsen [26]	Prospective Non-randomized controlled trial	Inclusion criteria: patients with osteoarthritis undergoing primary THA	365	191, 174	68.7	6 weeks
Mobasheri [27]	Prospective cohort	Inclusion criteria: patients under the age of 60 years	86	56, 30	51.4	3 years
Nevitt [28]	Retrospective cohort	Inclusion criteria: persons who were 60 years old or less who had a primary diagnosis of degenerative, congenital, or posttraumatic disease. Exclusion criteria: those who had severe, disabling articular problems other than in the hips prior to surgery and those with coexisting medical conditions prior to THA that might contribute to disability, such as heart disease, cancer, stroke, diabetes, and renal failure	178	78, 100	49.7	8–12 months
Oken [29]	Retrospective cohort	Inclusion criteria: patients with secondary osteoarthritis due to DDH aged < 60 years	51	7, 44	46.2	1 year
Pachore [30]	Prospective cohort	Inclusion criteria: patients with alkaptonuric hip arthritis	10	6, 4	62.8	16.7 years
Pagnano [31]	Prospective cohort	Inclusion criteria: patients with osteoarthritis (OA)	26	10, 16	69	6 months
Peak [32]	Prospective cohort	Inclusion criteria: patients without a history of surgery on the ipsilateral hip, hyper flexibility syndromes, and neuromuscular compromise	265	139, 126	58.3	6 months
Poehling- Monaghan [33]	Prospective cohort	Exclusion criteria: patients with a previous procedure on or around the operative femur or acetabulum, including prior arthroplasty, trauma, or corrective procedures	222	111, 111	64	2 months
Poehling-Monaghan [34]	Prospective cohort	Exclusion criteria: Patients if they had a prior procedure on the operative femur or acetabulum, prior trauma or infection to the area, inflammatory arthropathy, surgical intervention within the past 6 weeks	100	48, 52	63	8 weeks
Pons [35]	Retrospective cohort	Exclusion criteria: Patients with anatomic deformities in the femoral head and neck such as developmental dysplasia of the hip, slipped capital femoral epiphysis, post-septic and traumatic arthritis	128	90, 38	57.1	38.3 months
Pop [36]	Retrospective cohort	Inclusion criteria: patients who were under 65	32	18, 14	58	10 years
Rondon [37]	Prospective cohort	Exclusion criteria: Patients who underwent primary THA for hip fracture, revision TKA, or revision THA and those who were not employed or were retired before surgery	243		57.8	12 weeks
Saad [38]	Prospective cohort	Exclusion criteria: patients with severe protrusio or acetabular dysplasia and patients weighing more than 120 kg or body mass index (BMI) > 40	30	15, 15	59	6 to 8 months
Salar [39]	Retrospective cohort	Inclusion criteria: patients with acetabulum fracture	17	14, 3	52	48.2 months
Sankar [40]	Prospective cohort	Inclusion criteria: patients between 18 and 85 years of age with osteoarthritis. Exclusion criteria: revision or hemi-arthroplasty, and TJR for trauma or malignancy	190	100, 90	56.1	12 months
Stigmar [41]	Retrospective cohort	Inclusion criteria: patients with hip osteoarthritis resident in the Skåne region 1 year before surgery, and that they were 40–59 years old at the time of surgery. Exclusion criteria: Subjects who died or received disability pension/sickness compensation	1307	712, 595	53	2 years
Suarez [42]	Retrospective cohort	Exclusion criteria: patients under the age of 18 and over 64, housewives, students, and persons unemployed prior to treatment were excluded	747	598, 149	46.8	8 years
Takeuchi [43]	Retrospective cohort	Inclusion criteria: patients younger than 60 years of age. patients with primary THA due to osteoarthritis, avascular necrosis, or rheumatoid arthritis, which were treated with cementless implants. Exclusion criteria: patients with a history of postoperative complications (deep infection, fracture, dislocation, or revision for any reason), patients with extensive medical comorbidities that would limit their activity level, and patients who had had an osteotomy in the past	204	36, 168	53.8	59.5 months
Tanavalee [44]	Prospective cohort		70	28, 42	53.95	20.2 months
Tilbury [45]	Retrospective cohort	Inclusion criteria: patients with osteoarthritis, aged between 18 and 65 years, able to read and understand Dutch and being mentally and physically able to complete questionnaires. Exclusion criteria: patients with revision of a THA or TKA, undergoing a hemi arthroplasty and undergoing a THA or TKA because of tumor or rheumatoid arthritis	122	52, 70	57.7	1 year
Trusz czyń ska [46]	Retrospective cohort	Inclusion criteria: patients who are younger than 65. Exclusion criteria: patient not employed before THA (retired or on disability pension), other disorders preventing return to work, history of other surgeries, serious internal diseases, oncological diseases, infectious diseases of the musculoskeletal system	54	29, 25	55.89	23.52 months
Visuri [47]	Retrospective cohort		539	166, 373	63.87	4.2 years
White [48]	Retrospective cohort	Inclusion criteria: patients under 45 years of age	33	12, 21	38	7.5 years
Zaballa [49]	Retrospective cohort	Inclusion criteria: People underwent elective unilateral THA aged between 18 and 64 years and a minimum of 5 years had elapsed since their primary THA	411	206, 205	56	7.5 years

**Table 2:** The rate and time to return to work after total hip arthroplasty in the reviewed studies

Author	All patients	Patients not working before surgery (group 1)	Patients working before surgery (group 2)	All patient working after surgery	Patient working after surgery from group 1	Patient working after surgery from group 2	Median time to return to work	Other information
Al-Hourani [5]	91	27	64	62	8	54	Mean time to RTW following THA was 13.6 ± 7.5 weeks	–
Anderson [6]	23	0	23	22	0	22	mean days out of work after THA: 6-month follow-up: 90.8 (95% CI 69.9–111.8) 1-year follow-up: 100 (95% CI 67.8–132.2) 2-year follow-up: 115.9 (95% CI 54.5–177.2)	–
Atkinson [7]	–	–	–	–	–	–	Bilateral THA staged 1 week interval: Return to Work- Full time: 22 weeks Bilateral THA staged greater interval: Return to Work– Full time: 35.8 weeks	–
Baldursson [8]	10	–	–	6	–	–	–	–
Batra [9]	–	–	–	–	–	–	the average number of days taken to return to their usual work at any capacity was 24 days (range = 1–79 days)	–
Berger [10]	100	22	78	–	–	78	For the 78 patients who worked, was 8 days (range, 1–20 days)	–
Boersma [11]	68	0	68	61	0	61	Mean time to RTW of patients was 85 days (SD 69) following THA	Return to work (n (%)) 6 weeks full 7 (9%) 3 months full 25 (32%) 6 months full 51 (68%) 12 months full 61 (84%)
Bohm [2]	54	10	44	40	2	38	–	–
Clyde [12]	43	0	43	32	0	32	RTW time (wk) (95% CI) 17.3 (8.16–26.5)	–
Danielsson [13]	28	–	–	5	–	–	–	–
Drobniewski [14]	114	39	75	67	7	60	1.1–13.9 weeks	–
Drobniewski [15]	87	20	67	82	15	67	The mean length of sick leave was 196.2 days,	–
Goeb [16]	72	14	58	–	–	–	–	In this cohort, 10% of survey respondents had returned to work within 1 week of surgery. Twenty-four percent had returned by week 2, 40% by week 3, 74% by week 4, 75% by week 5, and 76% by 6 weeks postoperatively
Hauer [17]	267	–	–	242	–	–	Te median delay in RTW following THA was 10 weeks [IQR 7–14 weeks]	–
He [18]	128	41	87	98	15	83	–	By 3, 6, and 12 months after THR, 21, 46, and 31 of all the 128 patients returned to work, respectively
Jensen [19]	387	285	102	119	28	91	–	–
Johnsson [20]	118	–	–	69	–	–	196.68 days	–
Kamp [21]	–	–	–	–	–	–	days to return to work (median, IQR) 76.5 (49.0–113.5)	–
Kleim [51]	52	–	–	39	–	–	Weeks taken to return to work mean (SD) 12 (5.0)	–
Laasik [22]	408	–	408	383	–	383	After the surgery, 94% (n = 383) of the patients returned to work after a mean of 103 days (10–354) of sickness absence	–
Latijnhouwers [23]	–	–	–	–	–	–	–	Moreover, the proportion of PLA patients who had returned to work within 3 months after surgery was lower than in DAA patients (within 3 months: 31% vs. 44.6%, respectively). Whereas return to work 1-year after surgery was similar (86.2% and 84.9%, respectively, p¼0.815)
Leichtenberg [24]	67	0	67	53	0	53	–	–
Mangin [52]	72	–	72	45	–	45	mean (109 ± 91.67 days), (range, 15–515 days)	–
Mcgonagle [25]	–	–	–	–	–	–	Te average time of RTW was 6.4 ± 3.8 weeks for THA	–
Mikkelsen [26]	37	–	–	12	–	–	–	–
Mobasheri [27]	81	30	51	62	13	49	Patients working preoperatively took an average of 10.5 weeks to return to work. Patients not working preoperatively took an average of 35 weeks to gain employment	–
Nevitt [28]	139	58	81	95, 4 yrs later:(87)	20	75	–	–
Oken [29]	51	30	21	39	19	20	13.861 weeks	–
Pachore [30]	10	–	–	9	–	–	–	–
Pagnano [31]	–	–	–	–	–	–	39.85 days	–
Peak [32]	85	–	–	81	–	–	Finally, patients in the unrestricted group returned to work at a mean of 6.5 weeks whereas those in the restricted group returned at a mean of 9.5 weeks (p < 0.001)	–
Poehling-Monaghan [33]	71	0	71	59	0	59	–	–
Poehling-Monaghan [34]	100	–	–	57	–	–	Mean days (SD) 33 (21.8)	–
Pons [35]	128	55	73	–	–	70	–	–
Pop [36]	32	4	28	15, 10 years later:13	–	–	the average time to re – turn to work was between 1.1 and 13.9 weeks	–
Rondon [37]	243	0	243	221	0	221	5.56 weeks	–
Saad [38]	–	–	–	–	–	–	The mean value of time to rehab &return to work in the conventional approach group was 13.80 ± 4.50 weeks compared to 6.53 ± 0.72 weeks in the mini–invasive approach	–
Salar [39]	17	7	10	–	–	7	7.2 months (range: 1.5–24 months)	–
Sankar [40]	190	58	132	166	40	126	–	number of patients returned to work after: 1 months:65 3 months:124 6 months:161 12 months:166
Stigmar [41]	–	–	–	–	–	–	48 days sick leave after THR in two years follow	–
Suarez [42]	747	0	747	190	0	190	–	–
Takeuchi [43]	204	48	156	–	–	121	–	–
Tanavalee [44]	–	–	–	–	–	–	5 weeks	–
Tilbury [45]	119	51	68	67	3	64	the mean time to return to work was 12.5 weeks (SD 7.6; median 12; minimum 1; maximum 40 weeks)	–
Trusz czyń ska [46]	54	0	54	32	0	32	–	–
Visuri [47]	530	418	112	59	0	59	–	Return to work rate in males was 94.4%, which was significantly higher than that in females (52.3%)
White [48]	33	9	24	25	3	22	–	–
Zaballa [49]	784	303	481	506	57	449	–	–

### Preoperative working status and RTW rate

**Fig. 2:**
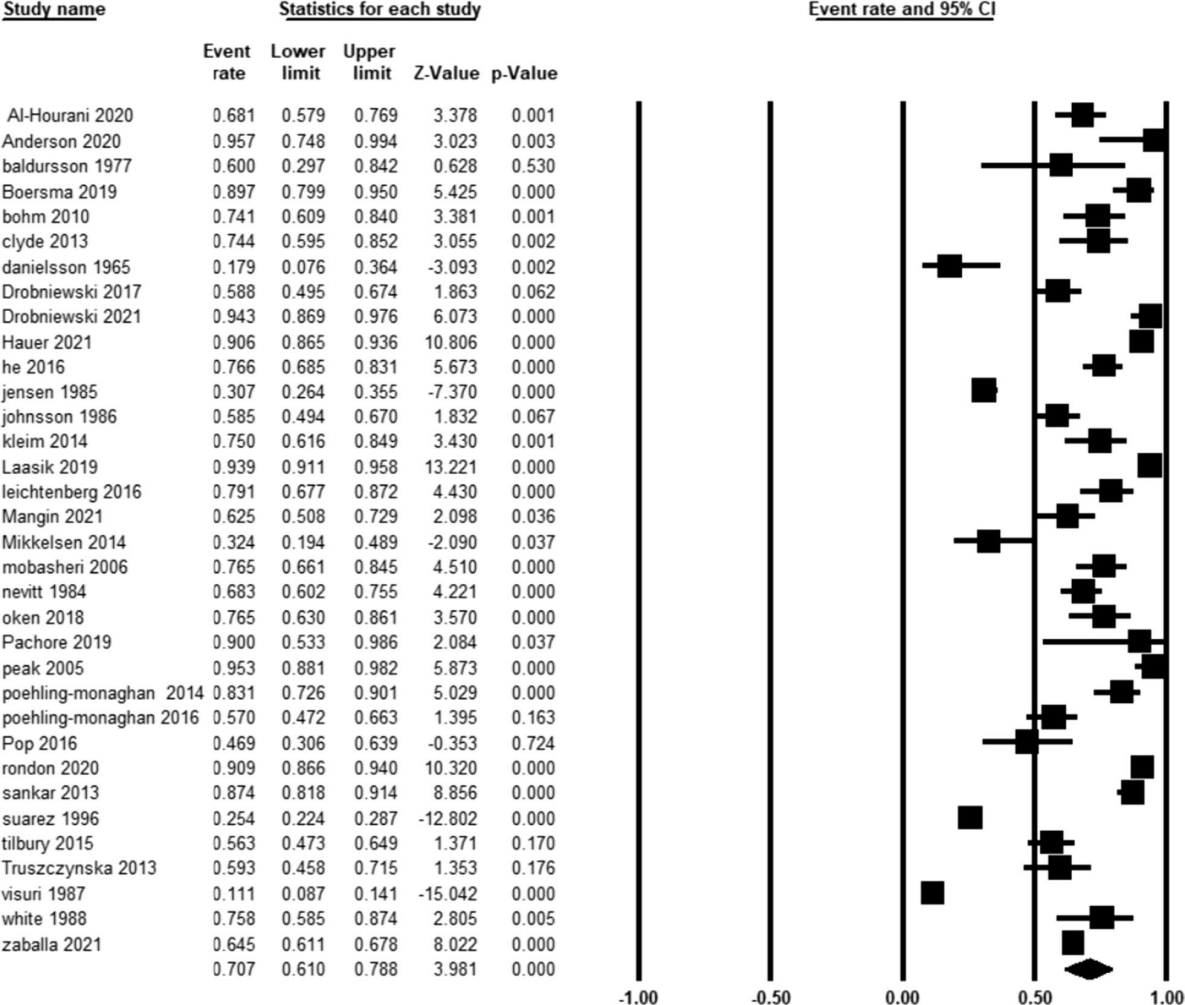
Ratio of patients working after THA in the selected studies

Table 2 summarizes the outcomes of included studies regarding working status before THA and RTW rates after primary THA. A total of 28 studies reported the preoperative working status, ranging from 21 to 100%. The same number of studies reported a rate of 11.1–95.7% for postoperative RTW (Fig. 2). The proportion of patients who working after THA compared to before THA ranged from 0.3 to 1.9.

**Fig. 3:**
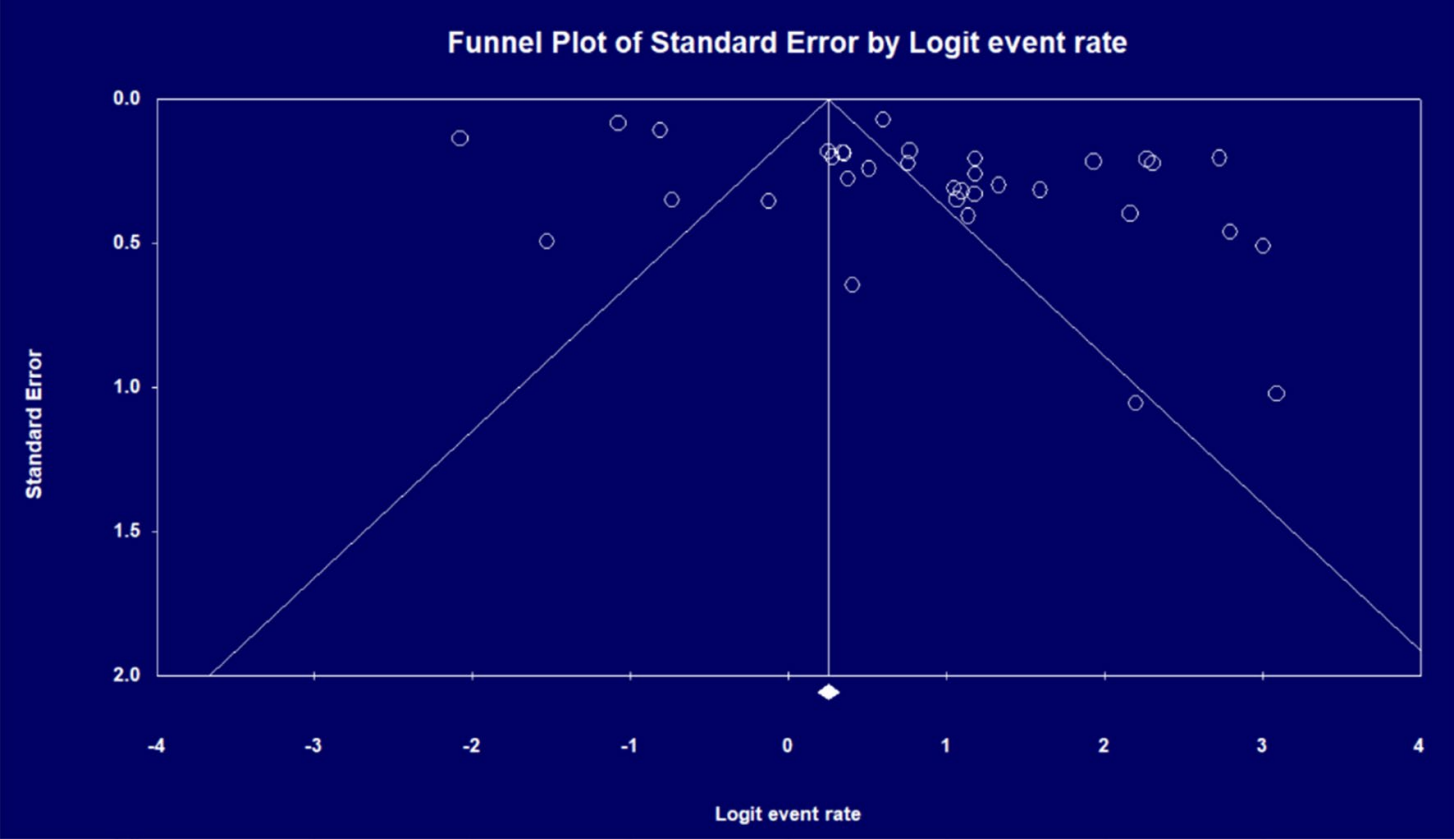
Funnel plot showing publication bias in the included studies

The pooled rate of working after THA in all patients using a random-effects model was 70.7% (95% CI = 61.0–78.8%, *I*^2^ = 97.4, Fig. 2). Figure 3 illustrates the funnel plot for publication bias with the Egger’s regression (*P* = 0.006).

**Fig. 4:**
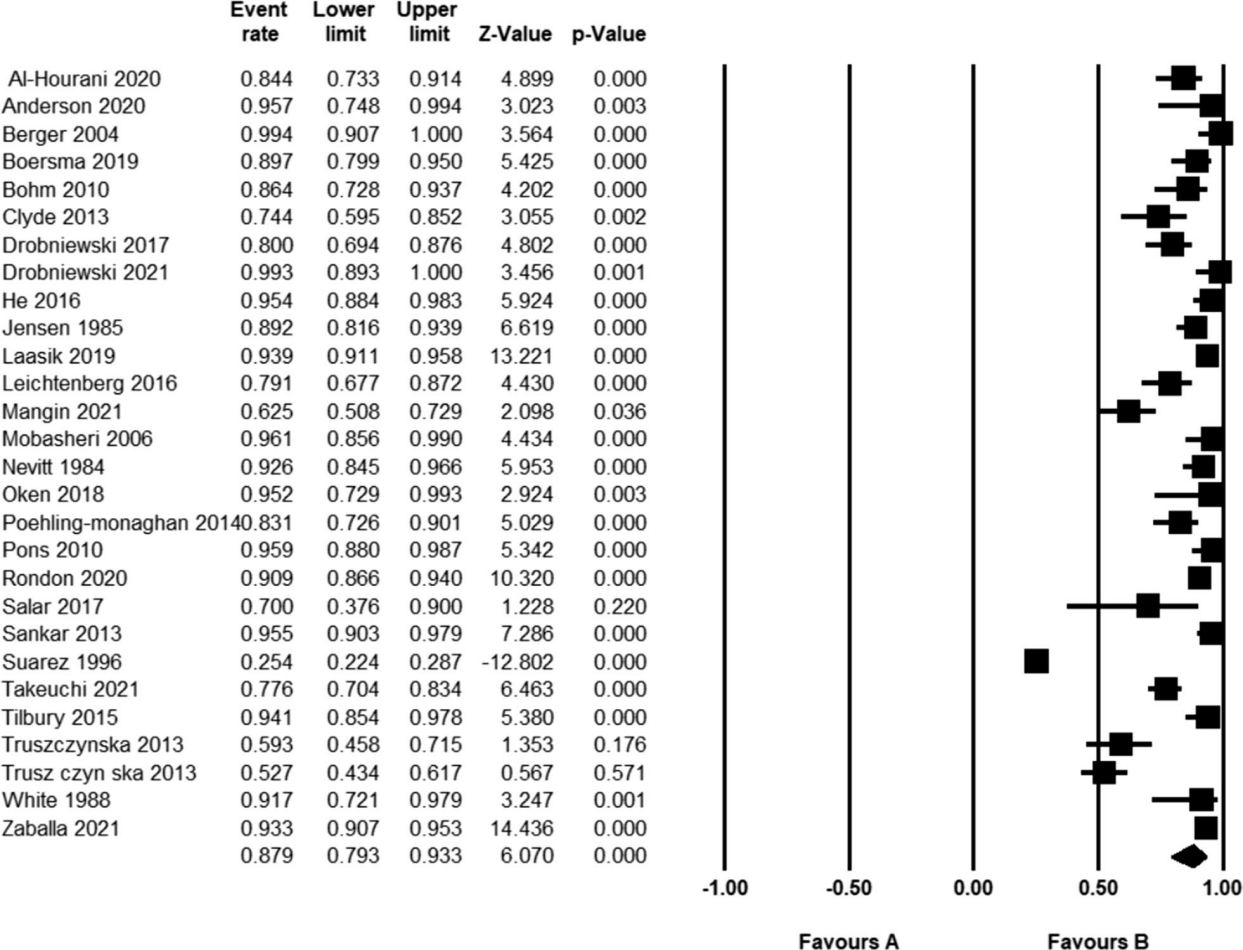
Rate of return to work after THA in patients who worked before surgery

The pooled rate of working after THA in patients who worked before, meaning a true RTW, using a random-effects model, was 87.9% (95% CI = 79.3–93.3%, *I*^2^ = 97.1, Fig. 4). The studies showed substantial publication bias both in the funnel plot and Egger's regression (*P* < 0.001).

**Fig. 5:**
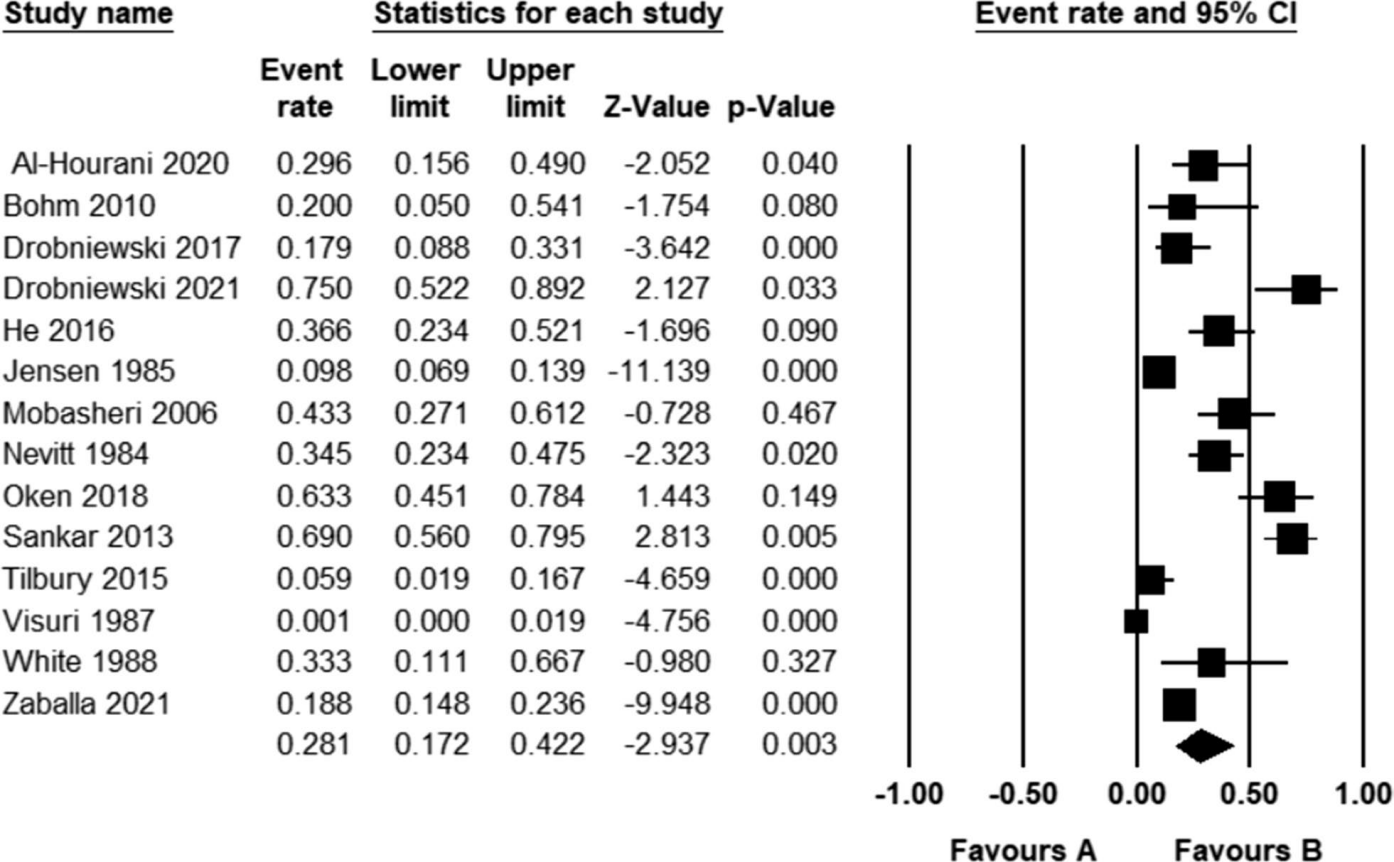
Percentage of non-working patients who started to work after THA

**Fig. 6:**
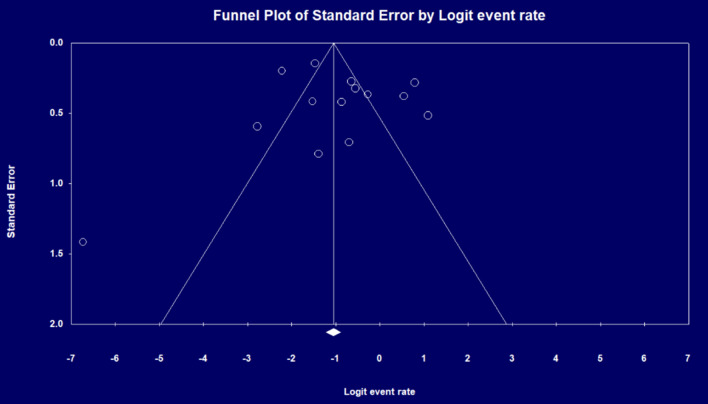
Funnel plot for publications bias in studies that reported “start to work” after THA

The pooled rate of working after THA surgery in the group of patients who did not work before that means “start to work”, using a random-effects model, was 28.1% (95% CI = 17.2–42.2%, *I*^2^ = 91.6, Fig. 5). The studies did not show substantial publication bias in the funnel plot (Fig. 6) and Egger's regression (*P* = 0.57).

### Quality of evidences

In assessing the quality of evidence based on GRADE system [53], “working after THA surgery in the group of patients who did not work” showed moderate quality of evidence, while other outcomes, “working after THA in all patients” and “working after THA in patients who worked before” showed low quality of evidence, and these results are justified by the type of included studies that were non-RCT besides the high level of heterogeneity in statistical analysis.

### Time to RTW after THA

Several studies reported mean time to RTW after THA, but the data were heterogeneous. The mean shortest time to RTW was 8 days (range 1–20 days) [10], with the mean longest duration to RTW of 31 weeks in one study reporting on patients with acetabular fractures [39].

#### Prognostic factors

*Preoperative intention to RTW* Only one study assessed the “intention to return to work” and found it to be a significant predictor of RTW after THA [25].

*Age* Four studies found a negative correlation between age and RTW [2, 24, 43, 47], while other studies did not find an association between RTW and age [12, 20, 22, 28, 29, 40].

*Time to RTW*: While three studies did not find a correlation between age and time to RTW [29, 37, 40], three other studies reported shorter time to RTW in younger patients [17–19].

*Gender* Six studies found male gender to be a significant predictor of RTW [2, 12, 20, 22, 28, 45], while other studies did not find this association significant [36, 43].

*Time to RTW*: Six studies reported the effect of sex on time to RTW, all reporting delayed RTW in female patients [17, 18, 27, 29, 37, 45].

*BMI* Patients who were not obese (BMI < 30) were found to be more likely to RTW in two studies [22, 29], but other studies did not find an association between RTW and BMI [2, 12, 24, 45].

*Time to RTW:* Oken et al. reported earlier RTW in non-obese (BMI < 30) patients [29, 36]. Two other studies reported no effect on the time to RTW [17, 37].

*Socioeconomic and education level* Only two studies showed that a higher educational level is associated with RTW [24, 42], while four other studies did not find an association [2, 28, 29, 46]. Pop et al. showed that living in a rural area was predictive for patients quitting their jobs after surgery [36].

*Time to RTW*: Three studies reported a negative correlation between education level and time to RTW [29, 37, 51], with patients having higher educational levels returning to work earlier (9.9 vs. 12.6 weeks, *P* < 0.05) [29, 51]. Other studies did not find such an association [17, 29]. Additionally, Oken et al. found that single patients RTW earlier (*P* < 0.05) [29].

*Substance use (smoking, alcohol, etc.)* Two studies reported no association between RTW and smoking or alcohol use [2, 22].

*Job characteristics* Mental and sedentary jobs, compared to physically demanding jobs, was associated with a higher rate of return to work in several studies [12, 13, 22, 42, 47]. One study reported patients with physically demanding jobs retiring after surgery [20]. However, other studies did not find an association between RTW and physical demand [2, 28]. McGonagle et al. [25] showed a higher probability of returning to work with reduced hours and duties among those with more physically demanding jobs. Self-employment was associated with partial or no return to work in one study (OR = 7.63, 95% CI 1.5–39.8) [24], while another study revealed no such association [2]. Zaballa et al. reported fewer patients returning to jobs that required prolonged standing, kneeling, squatting, or heavy lifting [49]. One study reported higher RTW rates when preoperative job satisfaction was lower [2].

*Time to RTW:* Several studies reported that workers with low-to-moderate physical demands, were more likely to RTW earlier [18, 21, 37, 51]. In contrast, two other studies showed no correlation [17, 25]. RTW was reported to be delayed in jobs requiring long durations of standing [37, 49]. Self-employment [17, 37] and higher income were associated with a shorter time to RTW [37].

*Indication for THA* Primary osteoarthritis, compared to posttraumatic osteoarthritis or rheumatoid arthritis, was associated with a higher rate of return to work [42, 47]. A history of developmental hip dysplasia was predictive of not RTW in two studies [14, 42].

*Time to RTW:* One study did not find the time to RTW to be associated with indications for surgery (osteoarthritis, avascular necrosis, hip dysplasia). However, among patients with underlying hip dysplasia/dislocation, Crowe Type 1 and 2 were found to have an earlier RTW compared to Crowe types 3 and 4 [29]. The same study reported earlier RTW in patients with smaller limb length discrepancy [29].

*Preoperative working status* Duration of the preoperative sick leave and absence from work were associated with a lower rate of RTW in one study (OR = 8.62, 95% CI 1.9–39.0) [24].

*Time to RTW:* Two studies reported that patients who were not working preoperatively took a longer time to RTW [18, 27]. We also found some evidence that preoperative sick leave delayed RTW [17, 22, 51].

*Preoperative functional status* One study found that a negative EQ-5D score preoperatively was a negative predictor of RTW after THA, and a preoperative Oxford hip score of > 19.5 was a predictor for RTW [5]. On the other hand, Leichtenberg et al. found that a higher HOOS-ADL score weakly predicted a partial or no return to work (OR = 1.03, 95% CI 1.0–1.1) [24]. EQ-5D and other patient-reported outcome measures (PROMs) were not associated with RTW in the study by Tillbury et al. [45].

*Time to RTW:* Time to RTW was not significantly associated with either total physical activity level or leisure-time physical activity [11, 22]. The use of a cane, crutch, or walker before surgery was not associated with delays to RTW [37].

*Postoperative functional status* Postoperative walking ability was significantly correlated with RTW in one study [47]. However, improvements in PROMs following THA were not found to be associated with RTW [5, 45, 47].

*Compared to similar procedures* Anderson et al. found that RTW for THA was significantly higher to internal fixation or hemiarthroplasty in the treatment of displaced femoral neck fractures [6]. The rate of patients returning to work following revision THA was also found to be lower than primary THA (43.9% vs. 70.2%, *P* < 0.05).

*Surgical approach and implants* Latijnhouwers et al. reported higher rates of RTW in THAs performed with a direct anterior approach compared to a posterolateral approach within the first 3 months of surgery (31% vs. 44.6%). However, the RTW rate was similar one year postoperatively (86.2% and 84.9%) [23]. In contrast, another study found a mini-posterior approach to have a higher rate of RTW 8 weeks after surgery (97% vs. 69%) [33]. One study found a higher rate of RTW with a larger femoral head diameter [43].

*Time to RTW:* A minimally invasive posterior approach resulted in a significantly shorter time to return to work compared to a conventional posterior approach in one study (6.5 vs. 13.8 weeks) [37]. Comparing traditional with minimally invasive approaches, Saad et al. revealed significantly lower time to RTW in the minimally invasive group (13.8 ± 4.5 vs. 6.5 ± 0.7, *P* < 0.001) [50]. Tanavalee et al. reported earlier RTW in patients who underwent a successful two-incision THA compared to a mini-posterior approach [44]. However, another study did not find a difference in time to RTW between surgical approaches [31]. Hauer et al. [17] did not find the stem design to affect the time to RTW (median 10 weeks with short stem compared to 11 weeks with straight stem, *P* = 0.7).

*Bilateral THA* Early return to work rate was higher in unilateral THA compared to bilateral THA [18]. Atkinson et al. reported that the time to return to full-time employment was significantly shorter in bilateral THAs staged 1 week apart compared to delayed two-stage surgeries (22.0 vs. 35.8 weeks, *P* = 0.02) without an increased risk for complications [7]. In contrast, Rondon et al. did not such an association [37].

*Postoperative restrictions* Two studies found postoperative range of motion restrictions to be predictive for failure to RTW, with over half of patients in the unrestricted group returning to work in 6 weeks, while only 18.8% [32] and 32% [26] of the restricted group returned to work during the same period.

*Time to RTW:* Same studies found a significant delay in RTW in the restricted groups (6.5 vs. 9.5 weeks, p 0.001) [25, 32].

*Serum markers* The levels of CPK, myoglobin, C-reactive protein, interleukin-6, and tumor necrosis factor-alpha were not predictive of RTW in one study [34].

## Discussion

This systematic review and meta-analysis was performed to determine the rate and time to RTW after primary THA in the current literature. The most important finding of this study was that the majority of patients return to work between 2 to 3 months after surgery. Furthermore, the rate of RTW seems to be increasing compared to previous systematic reviews. The results of this study are important in consulting patients of working age who need a THA, while also important from a healthcare economics standpoint.

In reviewing 48 studies with a combined population of 9267 patients, we found that 70.7% (95% CI = 61.0–78.8%) of patients were working after primary THA. Among patients who were working before surgery, this rate increases to 87.9% (95% CI = 79.3–93.3%). The previous systematic review, performed in 2017 by Hoorntje et al., reported 69% of patients working after THA, while 87.5% of patients working preoperatively returned to work. The increase in RTW and start-to-work rates from five years ago, while minimal, was seen in all ten newer studies published since 2017, with all ten papers reporting high rates of RTW than previously reported [9, 10, 17, 25, 31, 34, 37, 38, 41, 44]. This may be due to improved implant design, modern and minimally invasive surgical techniques, but may also be the result of increasingly younger patients, many of working age, are undergoing THA, emphasizing the importance of research on the topic. We also found that 28.1% (95%CI = 17.2–42.2%) of patients who were not working preoperatively started working after surgery, a finding not previously reported.

The studies reviewed here were not homogeneous in reporting factors predictive for RTW, but some conclusions could be made with the available data. Current suggests that younger age at surgery is associated with a higher rate of RTW [2, 24, 43, 47] and an earlier RTW [17–19]. Additionally, while some studies reported a delayed RTW in females compared to male patients [17, 18, 27, 29, 37, 45], the rate of RTW does not seem to be gender-related [2, 12, 20, 22, 28, 45]. Similarly, although overweight and obese patients experience delayed RTW [17, 37], the rate of RTW does not seem to be influences by BMI [2, 12, 24, 45]. Also, marital status and smoking and alcohol use were not predictive for RTW [2, 22]. However, the underlying hip pathology is important, with acetabular fractures and high-riding congenital hip dislocations having the longest RTW, exceeding 20 weeks in most reports [15, 39].

The job characteristics were also predictors of successful RTW after THA. Physically demanding jobs, and those requiring long-standing times seem to be the most important job characteristics, negatively influencing the rate of return and time RTW in several studies [12, 13, 18, 20–22, 37, 42, 47, 49, 51]. One study also found that providing a more flexible job schedule and reducing the physical demands of the job yield a higher rate of RTW, a luxury that is unfortunately not available for most patients [25]. Additionally, patients who missed work or had more sick leaves before surgery took a longer time to RTW, which may suggest that lower preoperative function would lead to a longer time to RTW [17, 18, 22, 27, 51].

We also found some surgical parameters influencing RTW. Minimally invasive THA and the direct anterior approach had a similar rate and speed of RTW [23, 37], superior to conventional posterior or lateral approaches [50]. Several studies showed that a minimally invasive approach resulted in faster recovery and early return to work following hip replacement surgery [50, 54–56]. However, implant design does not seem to be influential in RTW.

We acknowledge several limitations to this study. First, a high level of heterogenicity exists between the studies, population samples, and outcome measures involved. Second, limited by the lack of level I studies, we included retrospective and prospective cohorts as well, which makes the results more prone to bias. Third, some studies did not specify the preoperative working status of patients or the reason for not returning to work and whether it was related to surgery or not. Finally, several studies reported on a combination of THA and total knee patients, which may have different postoperative course and RTW. It was not possible to separate the data in some studies. One of the main limitations of our study is the difference in the surgical techniques considered for the patients that might be the cause of observed heterogenicity also because only limited number of studies focused minimally invasive surgeries expanding the results of this subject was not possible. Despite these limitations, we were able to provide a systematic update on the literature regarding the increasingly important topic of RTW after THA, while adding some new data to the literature. Fortunately an appropriate agreement was found between two authors searching the literature based on relevant keywords without any involving the third author for judgment.

We recommend to perform further studies with the focused on return to work considering special work condition, return to sport and physical activities and also assessing the factors influencing delayed return to work.

## Conclusion

This systematic review and meta-analysis showed that 70.7% of patients were working after THA. In patients who were working before surgery, this rate increases to 87.9%, while 28.1% of patients who were not working preoperatively started working after THA. Compared to previous reviews, patients seem to have a higher rate and earlier RTW. Younger patients were more likely to RTW, while patients with a physically demanding job were less likely to RTW. Minimally invasive techniques were reported to yield a higher rate of RTW and an earlier time to RTW. The overall trend of the literature suggests that patients are returning to work earlier and at a higher rate compared to previous systematic reviews. However, THA is a major surgery specially in elderly most patients returned to their work successfully and disability after surgery seem to be low. More interestingly, the feeling to ability to physical activities postoperatively led to tending of unemployed patients to seek the job and work actively.

## Data Availability

Not applicable.

## Appendix 1

See Table 3.

**Table 3:** Quality assessment based on the MINORS quality assessment

Study	A clearly stated aim	Inclusion of consecutive patients	Prospective collection of data	Endpoints appropriate to the aim of the study	Unbiased assessment of the study endpoint	Follow-up period appropriate to the aim of the study	Loss to follow-up less than 5%	Prospective calculation of the study size	An adequate control group	Contemporary groups	Baseline equivalence of groups	Adequate statistical analyses	Score
Al-Hourani [5]	2	2	2	2	0	2	2	0	2	2	1	2	19
Anderson [6]	2	2	0	2	0	2	2	0	2	2	0	2	16
Atkinson [7]	1	2	0	2	0	2	1	0	2	2	1	1	14
Baldursson [8]	1	0	0	2	0	2	2	0	N/A	N/A	N/A	N/A	7
Batra [9]	2	2	0	2	0	1	2	0	N/A	N/A	N/A	N/A	9
Berger [10]	1	2	2	2	0	1	2	0	N/A	N/A	N/A	N/A	10
Boersma [11]	2	2	2	2	0	2	1	0	2	2	1	2	18
Bohm [2]	1	2	0	2	0	2	1	0	2	2	1	2	15
Clyde [12]	2	2	2	2	0	2	1	0	2	2	2	2	19
Danielsson [13]	2	2	0	2	0	2	1	0	N/A	N/A	N/A	N/A	9
Drobniewski [14]	2	2	0	2	0	2	2	0	N/A	N/A	N/A	N/A	10
Drobniewski [15]	1	0	2	2	1	2	2	0	N/A	N/A	N/A	N/A	10
Goeb [16]	2	2	0	2	0	1	1	0	2	1	2	2	15
Hauer [17]	2	2	2	2	0	2	1	0	2	2	1	2	18
He [18]	2	2	2	2	0	2	2	0	2	2	1	2	19
Jensen [19]	2	2	0	2	0	2	2	0	2	2	1	1	16
Johnsson [20]	2	2	0	2	0	2	2	0	2	2	1	2	17
Kamp [21]	1	2	2	2	0	2	1	0	N/A	N/A	N/A	N/A	10
Kleim [51]	2	2	2	2	0	2	1	0	N/A	N/A	N/A	N/A	11
Laasik [22]	2	2	2	2	0	2	2	0	2	2	0	2	18
Latijnhouwers [23]	1	1	0	2	0	2	0	0	2	2	1	2	13
Leichtenberg [24]	2	2	2	2	0	2	1	0	2	2	2	2	19
Mangin [52]	2	2	2	2	1	2	1	0	2	2	2	2	20
Mcgonagle [25]	2	2	2	2	0	2	1	0	2	2	2	1	18
Mikkelsen [26]	1	0	2	2	1	1	1	2	2	1	1	2	16
Mobasheri [27]	2	2	0	2	0	2	1	0	N/A	N/A	N/A	N/A	9
Nevitt [28]	2	2	0	2	0	2	1	0	N/A	N/A	N/A	N/A	9
Oken [29]	2	1	0	1	0	2	2	0	2	2	2	2	16
Pachore [30]	1	1	2	2	0	2	2	0	N/A	N/A	N/A	N/A	10
Pagnano [31]	2	2	0	2	0	1	2	2	2	2	0	2	17
Peak [32]	2	2	2	2	2	1	2	2	2	2	2	2	23
Poehling-Monaghan [33]	2	2	0	2	2	1	2	0	2	2	2	2	19
Poehling-Monaghan [34]	2	2	1	2	0	1	1	2	2	2	1	2	18
Pons [35]	1	2	0	2	0	2	2	0	N/A	N/A	N/A	N/A	9
Pop [36]	2	1	0	2	0	2	2	0	N/A	N/A	N/A	N/A	9
Rondon [37]	2	2	0	2	0	2	2	0	2	2	2	2	18
Saad [38]	2	2	0	2	0	1	2	0	2	2	1	2	16
Salar [39]	1	2	0	2	0	2	2	0	N/A	N/A	N/A	N/A	9
Sankar [40]	2	2	0	2	0	2	2	0	2	2	2	2	18
Stigmar [41]	2	2	2	2	0	2	2	0	2	2	2	1	19
Suarez [42]	1	2	0	2	0	2	2	0	2	2	1	1	15
Takeuchi [43]	1	2	0	2	0	2	2	0	N/A	N/A	N/A	N/A	9
Tanavalee [44]	1	2	1	2	0	2	0	0	2	2	1	2	15
Tilbury [45]	2	2	2	2	1	2	2	0	2	2	2	2	21
Trusz czyń ska [46]	1	2	0	2	2	2	2	0	2	2	2	2	19
Visuri [47]	2	2	0	2	0	2	2	0	2	2	2	2	18
White [48]	1	2	0	2	0	2	2	0	N/A	N/A	N/A	N/A	9
Zaballa [49]	2	2	2	2	0	2	1	0	2	2	2	2	19
